# Renshen Baidu powder protects ulcerative colitis *via* inhibiting the PI3K/Akt/NF-κB signaling pathway

**DOI:** 10.3389/fphar.2022.880589

**Published:** 2022-08-10

**Authors:** Zhen Ye, Yuzheng Li, Yingqi She, Mingquan Wu, Yu Hu, Kaihua Qin, Linzhen Li, Han Yu, Qian Zhao, Zhao Jin, Fating Lu, Qiaobo Ye

**Affiliations:** ^1^ School of Basic Medical Sciences, Chengdu University of Traditional Chinese Medicine, Chengdu, China; ^2^ Department of Pharmacy, Sichuan Orthopedic Hospital, Chengdu, China; ^3^ Health Preservation and Rehabilitation College, Chengdu University of Traditional Chinese Medicine, Chengdu, China

**Keywords:** Renshen Baidu powder, ulcerative colitis, PI3K, Akt, NF-κB, TNBS-induced colitis

## Abstract

Ulcerative colitis is a chronic and relapsing inflammatory bowel disease without satisfactory therapy available recently. Renshen Baidu powder (RSBDP) is a classic Chinese medicinal formula used since Chinese Song dynasty and has been proven as an effective treatment of ulcerative colitis in clinics. However, the active ingredients and the molecular mechanism have not been fully disclosed. It is imperative to explore the active ingredients and the mechanism of RSBDP. In this study, the potential active components for ulcerative colitis treatment in RSBDP were determined and predicted in silicon, and its molecular mechanisms were also presented, in which the PI3K/Akt/NF-κB signaling pathway was recognized to be vital. Basically, the pharmacodynamics and mechanistic studies of RSBDP for ulcerative colitis were implemented on TNBS-induced experimental rats. The results showed that RSBDP could ameliorate the disease activity index and colon weight, as well as improve colonic shortening and colon histology. In addition, the tumor necrosis factor-α (TNF-α), diamine oxidase, intercellular adhesion molecule-1, and endotoxin in serum were also reduced. It is worth mentioning that the PI3K/Akt/NF-κB signaling pathway was inhibited after RSBDP administration *via* inhibiting the phosphorylation of proteins. In conclusion, RSBDP effectively ameliorates TNBS-induced colitis rats by inhibiting the PI3K/Akt/NF-κB signaling pathway.

## 1 Introduction

Ulcerative colitis (UC) is a recurring, chronic immunological illness, which is classified as a subtype of inflammatory bowel disease (IBD). This disease is directly related to the major changes in human society, transportation, diet, agriculture, manufacturing, and urbanization that occurred in Europe throughout the 19th century as a result of industrialization (Kaplan, 2015). Industrialized regions such as Europe, North America, and Oceania have historically maintained a high rate of incidence and prevalence. Global industrialization and urbanization accelerated the pace of expansion of IBD incidence in newly industrializing regions (Ordás et al., 2012; Kaplan and Ng, 2016). In 2011, the average incidence of IBD in Asia was 1.4 per 100,000. In the recent decade, the incidence of IBD has risen considerably in Asia. In addition, the incidence of UC is twice as high as that of Crohn’s disease in Asia. At the same time, experts believe that the prevalence of UC has not improved due to the absence of an ideal cure. In addition, the development of global aging society causes those elderly patients to be the largest growing group of IBD patients (Faye and Colombel, 2022). Kaplan and Windsor believed that the number of elderly IBD patients will increase in the next decade (Windsor and Kaplan, 2019). The severe condition demonstrates that UC is a significant public health problem that has imposed a significant economic and health burden on the international society. The pathological injury of UC mainly involves the mucosal and submucosal layers of the colon. The lesions mostly involve the rectum and extend proximally to the colon, while a small proportion of severe UC or fulminant UC may present with penetrating injury and pathological injury to the whole intestinal segment. An abnormally hyperactive immune response is thought to be one of the major pathological mechanisms of UC. Based on the characteristics of the disease, the current clinical first-line therapies include aminosalicylates (5-ASA), corticosteroids, immunosuppressants (e.g., thiopurines), and surgical treatment. However, most limits have been pointed out. Furthermore, 5-ASA is beneficial to mild to moderate UC but ineffective to severe UC. Meanwhile, corticosteroids are considered to lack long-term efficacy and safety (Ko et al., 2019). Immunosuppressants are thought to increase the risk of infection. Also, Windsor et al. (2013) mentioned that surgery could delay the progression to some extent, but the prognosis of surgery still carries the risk of colon cancer (Windsor et al., 2013). In essence, satisfactory therapy remains an enigma. There is an urgent need for the development of more safe and effective therapy solutions.

The Renshen Baidu powder (RSBDP) composed of *Panax ginseng* C. A. Mey. (Renshen, RS), *Poria cocos* (Schw.) Wolf (Fuling, FL), *Glycyrrhiza uralensis* Fisch. ex DC. (Gancao, GC), *Citrus* × *aurantium* L. (Zhiqiao, ZQ), *Platycodon grandiflorus* (Jacq.) A. DC. (Jiegeng, JG), *Bupleurum chinense* DC. (Chaihu, CH), *Kitagawia praeruptora* (Dunn) Pimenov (Qianhu, QH1), *Hansenia weberbaueriana* (Fedde ex H.Wolff) Pimenov & Kljuykov (Qianghuo, QH2), *Angelica biserrata* (R.H. Shan & C.Q. Yuan) C.Q. Yuan & R.H. Shan (Duhuo, DH), and *Conioselinum anthriscoides* “*Chuanxiong*” (Chuanxiong, CX) was first documented in Beneficial Formulas from the *Taiping Imperial Pharmacy* (*Tài Píng Huì Mín Hé Jì Jú Fāng*). This classical medicinal formula embodies the traditional Chinese treatment method of “rowing the boat against the stream”. By using eliminating pathogenic medicinals, the directed pathogens that cause intestinal diseases are dispelled from the superficial intestinal level, as if pulling a boat upstream (Xu et al., 2021). The medicinal formula has been widely applied to treat patterns of dysentery caused by external pathogens that have penetrated the interior. Research showed that RSBDP has anti-inflammatory, anti-infective, and mucosal healing properties. This medicinal formula has been widely applied to gastrointestinal diseases in clinical practices. According to the article from Zhou et al. (2014), the total efficacy rate of RSBDP treating irritable bowel syndrome clinically reached 84.9% (Zhou et al., 2014). Meanwhile, RSBDP was demonstrated to inhibit 2,4,6-trinitrobenzene sulfonic acid (TNBS)–induced colitis in rats by downregulating IL-1, IL-6, TNF-α, and IFN-γ; upregulating colonic mucosal occludin, claudin-5, and ZO-1 mRNA protein expression; and promoting the repair of tight junction protein colonic epithelium (Xiong et al., 2021). However, the deeper process of RSBDP inhibiting colon inflammatory activity has not been explicitly characterized or indicated. Meanwhile, the multi-component and multi-target mechanisms of RSBDP on UC have not been fully revealed.

Network analysis has the advantage of integrating multi-component and multi-target data. This method can explore the network regulation mechanism of multiple compounds and multiple targets (Zhang et al., 2019). The method can reveal the importance of medicinals combination of RSBDP for UC and give some purposeful research evidences rapidly. Meanwhile, the combination of the network analysis and animal experiment validation can provide a comprehensive evidence chain of RSBDP for UC. Therefore, this study aims to reveal a molecule–target network regulation mechanism of RSBDP on UC using the network analysis and biological investigation. More importantly, this study provides the basic evidence for a clinical protocol.

## 2 Materials and methods

### 2.1 Network construction of Renshen Baidu powder putative target and the ulcerative colitis gene

The corresponding targets of RSBDP were obtained through target prediction and functional analyses of TCM (including medicinal formulas) using the Integrative Pharmacology-based Research Platform of Traditional Chinese Medicine (TCMIP V2.0 http://www.tcmip.cn/TCMIP/index.php) database, which is based on ETCM, an Encyclopedia of Traditional Chinese Medicine (http://www.nrc.ac.cn:9090/ETCM/) (Xu et al., 2017; Xu et al., 2019). The search object was limited to Chinese medicine, and the keywords were “Renshen,” “Fuling,” “Gancao,” “Zhiqiao,” “Jiegeng,” “Chaihu,” “Qianhu,” “Qianghuo,” “Duhuo,” and “Chuanxiong.” The principle underlying target prediction uses MedChem Studio (version 3.0) software to search the DrugBank database for structural similarities between the two-dimensional structures of chemical components and the certified drug (Approved), followed by scoring the similarity using the Tanimoto coefficient. When the similarity score was ≥ 0.8 (moderate to high similarity), the potential targets for the RSBDP were obtained.

To investigate the relationship between RSBDP putative targets and ulcerative colitis genes, a list of ulcerative colitis–related genes were also collected from the disease-related gene database of TCMIP, which integrates Human Phenotype Ontology (HPO, https://hpo.jax.org/app/), Online Mendelian Inheritance in Man (OMIM, https://www.omim.org/), Therapeutic Target Database (TTD, http://db.idrblab.net/ttd/), DrugBank (https://go.drugbank.com/), DisGeNET (https://www.disgenet.org/), and Orphanet (https://www.orpha.net/consor/cgibin/index.php). The keyword for ulcerative colitis was “ulcerative colitis”. An interaction network of RSBDP putative target and ulcerative colitis–related gene was constructed based on the links among the two gene sets using the TCM Association Network Mining (TCMNM) module of TCMIP, which directly exhibits the major hub network according to three topological features of each node gene, including “degree,” “betweenness,” and “closeness”.

### 2.2 Network visualization and functional enrichment analysis

To better exhibit the common targets among RSBDP and UC, a Venn diagram was prepared using the software Tbtools (Chen et al., 2020). Also, to better display the interactions of the major hubs, network visualization was performed using Cytoscape 3.8.0. The biological functions and participated pathways of the major hubs were investigated by TCMIP V2.0, which is according to the Gene Ontology (GO) and the Reactome pathway (https://reactome.org/) (Xu et al., 2019).

### 2.3 Preparation and identification of the primary compounds of Renshen Baidu powder

**FIGURE 1:**
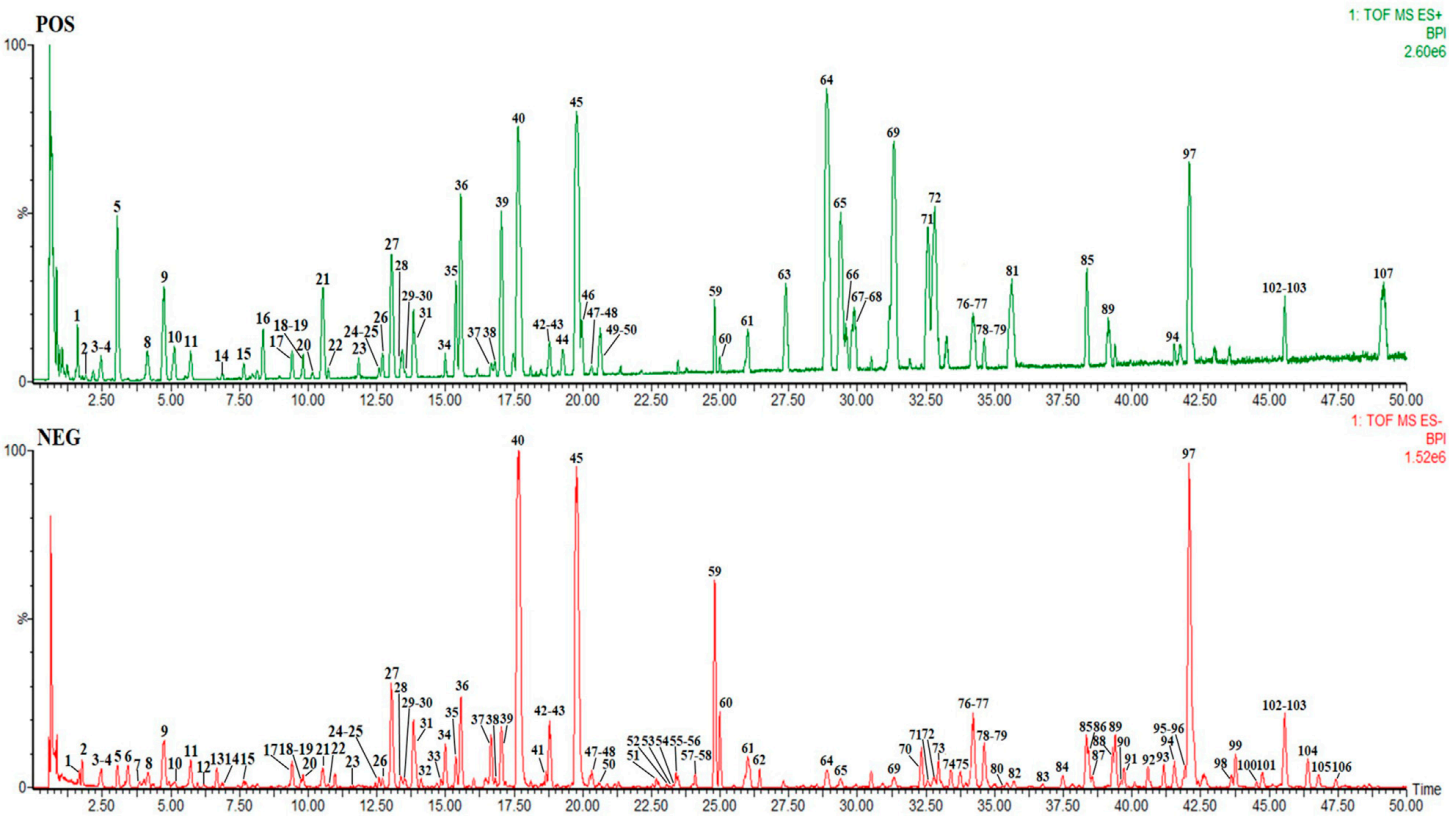
Base peak ion chromatograms of water extracts of RSBDP.

RSBDP, composed of *Panax ginseng* C. A. Mey. (Araliaceae; *Panax ginseng* radix et rhizoma] (Renshen, RS), *Poria cocos* (Schw.) Wolf (Polyporaceae; *Poria cocos* radix et rhizoma) (Fuling, FL), *Glycyrrhiza uralensis* Fisch. ex DC. (Fabaceae; *Glycyrrhiza uralensis* sclerotia) (Gancao, GC), *Citrus × aurantium* L. (Fabaceae; *Citrus × aurantium* fruit) (Zhiqiao, ZQ), *Platycodon grandiflorus* (Jacq.) A. DC. (Fabaceae; *Platycodon grandiflorus* radix) (Jiegeng, JG), *Bupleurum chinense* DC. (Apiaceae; *Bupleurum chinense* radix) (Chaihu, CH), *Kitagawia praeruptora* (Dunn) Pimenov (Apiaceae; *Kitagawia praeruptora* radix) (Qianhu, QH1), *Hansenia weberbaueriana* (Fedde ex H. Wolff) Pimenov & Kljuykov (Apiaceae; *Hansenia weberbaueriana* radix et rhizoma) (Qianghuo, QH2), *Angelica biserrata* (R.H. Shan & C.Q. Yuan) C.Q. Yuan & R.H. Shan (Apiaceae; *Angelica biserrata* radix) (Duhuo, DH), and *Conioselinum anthriscoides* “*Chuanxiong*” (Apiaceae; *Conioselinum anthriscoides* “*Chuanxiong*” rhizoma) (Chuanxiong, CX), was purchased from Sichuan Xinhehua Traditional Chinese Medicine Co., Ltd. Material authentication for TCM identification was carried out by Prof. Wei Liu of Chengdu University of Traditional Chinese Medicine. The voucher specimen (No. 20210305-1 for *Panax ginseng* C. A. Mey., No. 20210305-2 for *Poria cocos* (Schw.) Wolf, No. 20210305-3 for *Glycyrrhiza uralensis* Fisch. ex DC., No. 20210305-4 for *Citrus × aurantium* L., No. 20210305-5 for *Platycodon grandiflorum* (Jacq.) A. DC., No. 20210305-6 for *Bupleurum chinense* DC., No. 20210305-7 for *Kitagawia praeruptora* (Dunn) Pimenov, No. 20210305-8 for *Hansenia weberbaueriana* (Fedde ex H. Wolff) Pimenov & Kljuykov, No. 20210305-9 for *Angelica biserrata* (R.H. Shan & C.Q. Yuan) C.Q. Yuan & R.H. Shan, and No. 20210305-10 for *Conioselinum anthriscoides* “*Chuanxiong*”) was deposited by Qiaobo Ye at the Institute of Basic Research in Clinical Medicine of Chengdu University of Traditional Chinese Medicine (Chengdu, China). RS, FL, GC, ZQ, JG, CH, QH1, QH2, DH, and CX were mixed at a 5:5:3:5:5:5:5:5:5:5 ratio to obtain 1,440 g of raw materials. Medicinal materials were immersed in 11,520 ml of distilled water for 1 h, boiled with a high heat until boiling and turned into a low heat for 30 min, and then filtered with a 400 mesh filter cloth. Herb residues were again soaked in 11,520 ml water, boiled for 1 h, and filtered again. Both filtered decoctions were combined and concentrated with rotary evaporation at 60–80°C. After evaporation, 1440 ml of extract was obtained, where 1 ml of the extract contained 1 g of the raw materials, and it was stored at 4°C. The requirements considered relevant in recent best practice guidelines for pharmacological studies of natural products have been taken into account (Heinrich et al., 2020). Waters Acquity UPLCTM ultra-high performance liquid chromatography was used for identifying compounds’ composition of RSBDP. The mass spectrum of RSBDP is shown in Figure 1, and the identification of chemical compounds in RSBDP by UPLC-Q-TOF/MS is shown in Supplementary Table S1. The Traditional Chinese Medicine Systems Pharmacology Database and Analysis Platform (TCMSP) was used to obtain oral bioavailability (OB) and drug-likeness (DL) of the compounds (Ru et al., 2014). The test solution was centrifuged at 13000 rpm for 15 min, and the supernatant was taken and filtered through a 0.22-μm microporous membrane. Waters Acquity UPLCTM ultra-performance liquid chromatography was selected, including a quaternary solvent management system, sample management system, PDA detector, and empower chromatographic workstation (Waters, United States). The Q-TOF analyzer of the SYNAPT G2 HDMS system, ESI interface, and data processing system for MarkerLynx 4.1 workstation were used (American Waters Company). Sorvall Legend Micro 17 high-speed centrifuge (Thermo Fisher Scientific, United States), BT125D 100,000th electronic balance (Sartorius, Germany), KQ-500E ultrasonic cleaner (Jiangsu Kunshan Ultrasonic Instrument Co., Ltd.), microfluidic gun (Thermo Scientific, United States), and chromatographic column: Acquity UPLCR BEH C18 (2.1 mm × 100 mm, 1.7 μm) column were utilized. The mobile phase was 0.1% formic acid water (A) and 0.1% formic acid in acetonitrile (B), flow rate was 0.4 ml·min^−1^, column temperature was 40°C, and injection volume was 1 μl. The gradient elution program is as follows: 0–3 min 95% A, 3–30 min 74% A, 30–35 min 72% A, and 35–50 min 62% A. Mass spectrometry wad performed using the Waters SYNAPT G2 HDMS system. Nitrogen was used as atomization and cone-hole gas; source temperature (Source temperature): 150°C, reverse cone-hole gas flow (Cone gas flow): 50 L/h, desolvation temperature (Desolvation temperature): 450°C, desolvation gas flow (Desolvation gas flow): 800L/h, sample cone voltage (Sampling): 40 V, extraction cone voltage (Extraction cone): 4 V, capillary voltage (Capillary voltage): positive mode 3.0 kV and negative mode 2.5 kV, scanning time (Scan time): 0.3 s, scanning interval (Interscan time): 0.02 s, mass-to-charge ratio: m/z 100–1700 Da, and locking mass number (leucine enkephalin): positive ion mode: m/z 556.2771 (M + H)+ and negative ion mode: m/z 554. 2615 (M−H]−.

### 2.4 Animal experiment

**FIGURE 2:**
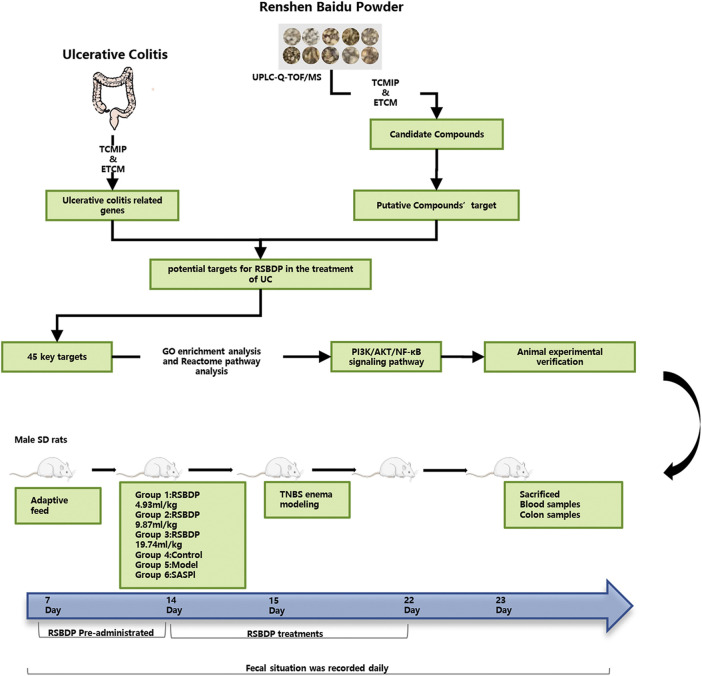
Scheme of the present study.

A total of 72 SD male rats weighing 180–220 g were purchased from Dashuo (Dashuo Co., Chengdu, Sichuan, China). Before the experiment, rats were fed adaptively for 7 days in the environment of room temperature 20–23°C, humidity 50%–60%, and natural dark cycle. Rats were randomly divided into a normal control group, model group, positive drug sulfasalazine control group (SASP), high-dose RSBDP group (RSBDPH), medium-dose RSBDP group (RSBDPM), and low-dose RSBDP group (RSBDPL), with six rats in each group. During the study, laboratory animals were provided with food and water *ad libitum*. These rats were placed in the Laboratory Animal Research Center of Chengdu University of Traditional Chinese Medicine approved by Sichuan Laboratory Animal Care Certification Association. Dosage of RSBDP and SASP were determined based on the conversions from clinical adult dosages. The dosage of RSBDP for adult was 96 g (the total raw materials)/day; equivalently, the dosage for rat was 9.87 mg/kg/day calculated by the formula that converts dosage of human into that of rat according to the respective body surface areas in accordance with the Research Methods in Pharmacology Chinese Materia Medica (Chen et al., 2011). Therefore, this study set 4.93, 9.87, and 19.74 g/kg/day RSBDP as the low, middle, and high dose in rats, respectively. The dose of the sulfasalazine group was 360 mg/kg/day. Except for the normal control and the model groups, all rats were pre-treated after adaptive feeding for 7 days. Except for the normal control group, all rats were anesthetized by Zoletil (Virbac SA Co., Carros, France) on the 15th day with 5% TNBS (Sigma Chemical Co., St. Louis, MO, United States) mixture (5% TNBS solution and 95% ethanol solution were mixed at a ratio of 1:1). The model was established by enema according to 100 mg/kg weight and then fed normally. The rats were fasted 36 h prior to model establishment without water deprivation. Consolidate on the fourth day of the modeling. The rats were anesthetized, and the hose was slowly pierced into the anus and TNBS was injected, while the control group was injected with the same amount of 9% sodium chloride solution. Take a low head and high tail position during the injection, and keep 15–30 min in this position. After the model was established, the rats lay flat and awake naturally, and then were fed routinely. Dosing cycle lasted 7 days. The control group and model group rats were given the same amount of 0.9% sodium chloride solution. The experimental period was of 22 days; after 23rd day, all rats were sacrificed, and blood and colon samples were taken. The body weight, stool characteristics, and fecal blood score of rats were recorded daily, and the DAI (disease activity index) was performed during the experiment (Figure 2).

### 2.5 Evaluation of disease activity index

Colitis was quantified based on DAI scores of body weight, stool consistency, and gross bleeding. DAI scoring details are shown in Supplementary Table S2. In brief, an investigator complying with the protocol recorded and scored the changes in weight, hemoccult positivity or gross bleeding, and stool consistency according to the previous report.

### 2.6 Histopathology

The colon tissue of rats was immediately dissected out and the length was measured with a ruler. After that, the tissue was processed in the form of Swiss rolls, fixed with 4% paraformaldehyde, and followed by gradient ethanol dehydration, xylene transparency, paraffin embedding, tissue sectioning, and HE staining. Then the pathological damage of the colon tissue was observed under light microscopy.

### 2.7 Enzyme-linked immunosorbent assay

After being treated with RSBDP, whole bloods samples were collected from blood circumcision after reperfusion. Immediately, blood samples were centrifuged at 12,000 g for 10 min at 4°C, and then the supernatant was collected for measurement at −20°C. The levels of tumor necrosis factor-α (TNF-α), intercellular adhesion molecule-1 (ICAM-1), endotoxin (ET), and DAO were detected with corresponding ELISA kits (Jiangsu Jingmei Biotechnology company, China). The whole experiment procedure was carried out according to the operational protocol.

### 2.8 Real-time PCR analysis

Total RNA of colon was obtained according to the RNAiso Plus reagent (9109, Takara, China) instructions. cDNA was synthesized according to the instruction of Transcriptor First Strand cDNA Synthesis Kit (04,897,030,001, Roche, German). Reverse transcription was performed at 55°C for 30 min in a mixture containing control RNA, anchored-oligo (dT)18 primer, RNase Free dH2O (RT-121, Tiangen Biotech, China), transcriptor reverse transcriptase reaction buffer, protector RNase inhibitor, deoxynucleotide mix, and transcriptor reverse transcriptase. The mixture was heated at 85°C for 5 min to inactivate the reverse transcriptase to obtain cDNA. Real-time PCR was performed using the Stormstar SYBRGreen qPCR Master Mix (DBI-2143, DBI Bioscience, Germany) and detected by CFX96 with a 20 ul system (BioRad, United States). The primer sequences were 5′-TGG​ACG​ATC​TGT​TTC​CCC​TC-3’/5′-CCC​TCG​CAC​TTG​TAA​CGG​AA-3′ for NF-κB, 5′-GCA​GGA​GTG​TTG​GTG​ACT​GA-3’/5′-CTG​AGG​CAT​CTC​TTG​GGT​GG-3′ for IκB, 5′-CAG​ACG​GAG​TTT​GGC​ATC​AC-3’/5′-TCG​GGC​TCC​TCT​GTA​GGT​C-3′ for IKKβ, 5′-TACGGTGCGGAGATTGTGTC-3’/5′-GCACCGTCCTTGATACCCTC-3′for Akt, and 5′-ACT​GTA​TTT​GTG​GGA​GCG​GT-3’/5′-CGC​AAG​AAA​GAT​GCC​TTG​TGT-3′ for PI3K.

### 2.9 Western blot analysis

The protein sample was added to the corresponding proportion of the SDS gel loading buffer and boiled for 5 min. After SDS-PAGE and transferring the protein on the membranes, the membranes was blocked with 5% skim milk in the PBST buffer at room temperature for 1 h and washed with PBST three times. Then β-actin, PI3K, Akt, NF-κB, p-Akt (β-Actin (13E5) Rabbit mAb #4970, #4257, #4691, #8242, and #13038, Cell Signaling Technology, Danvers, MA, United States), p-PI3K, and p-NF-κB (ab182651, EP2294Y, Abcam, Cambridge, MA, United States) (1:500) primary antibodies were added, and the membranes were incubated overnight at 4°C, washed three times with PBST, and incubated in the corresponding secondary antibodies linked with HRP (anti-rabbit IgG, HRP-linked antibody #7074). Then on the membranes were added radish peroxidase and incubated for 1 h at room temperature. The membranes were washed three times, and ECL was used. All intensities of the protein bands were detected by the ChemiDOCTM MP-imaging system; the gel imaging system was photographed, and the ImageJ (National Institutes of Health, Bethesda, MA, United States) was used to analyze the gray value.

### 2.10 Statistical analysis

Data were expressed as mean ± standard error of the mean (X ± SEM). GraphPad Prism 9 was used for the statistical analysis, while one-way analysis of variance was used for comparison between groups. The difference was statistically significant at *p* < 0.05. All data were plotted by using GraphPad Prism 9.

## 3 Results

### 3.1 Putative targets of ulcerative colitis treated by Renshen Baidu powder

**FIGURE 3:**
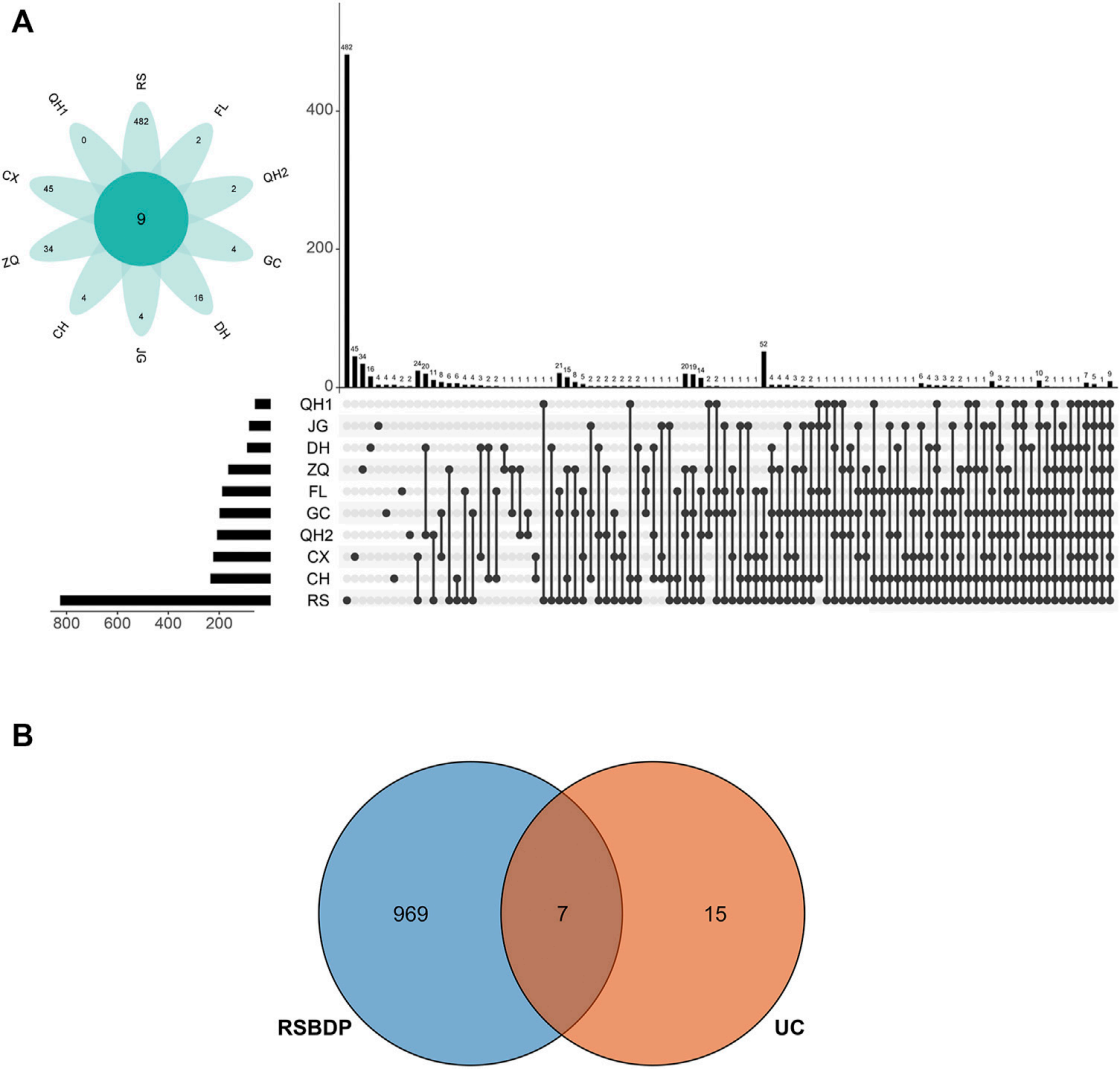
Putative targets of RSBDP on UC. **(A)** UpSetR plots depicting the number of unique and shared differentially putative targets among every medicinals. The top number above each bar represents the number of putative targets that are unique to each medicinal combination. The medicinal combination is indicated by linked dots below the *x*-axis. In addition, the flower plot shows number of unique putative targets (in the petals) and common putative targets for all medicinals (in the center); **(B)** Venn diagram of RSBDP putative targets and known UC-related genes.

A total of 981 putative targets were predicated through the TCM Target Prediction and Function Analysis Module of TCMIP. The putative targets amount of RS, QH1, CX, ZQ, CH, JG, DH, GC, QH2, and FL were 823, 57, 221, 162, 232, 80, 88, 196, 206, and 186, respectively (Supplementary Table S3). Interestingly, these 10 medicinals share several putative targets according to the prediction, indicating the potential medicinal–medicinal interactions through their shared targets (Figure 3A). Also, according to the disease-related gene database in TCMIP, the present study identified 22 UC-related genes (Supplementary Table S4). There are seven shared targets between UC and RSBDP (Figure 3B), indicating that these targets may be the possible effects targets.

### 3.2 Major mechanisms of Renshen Baidu powder on ulcerative colitis

**FIGURE 4:**
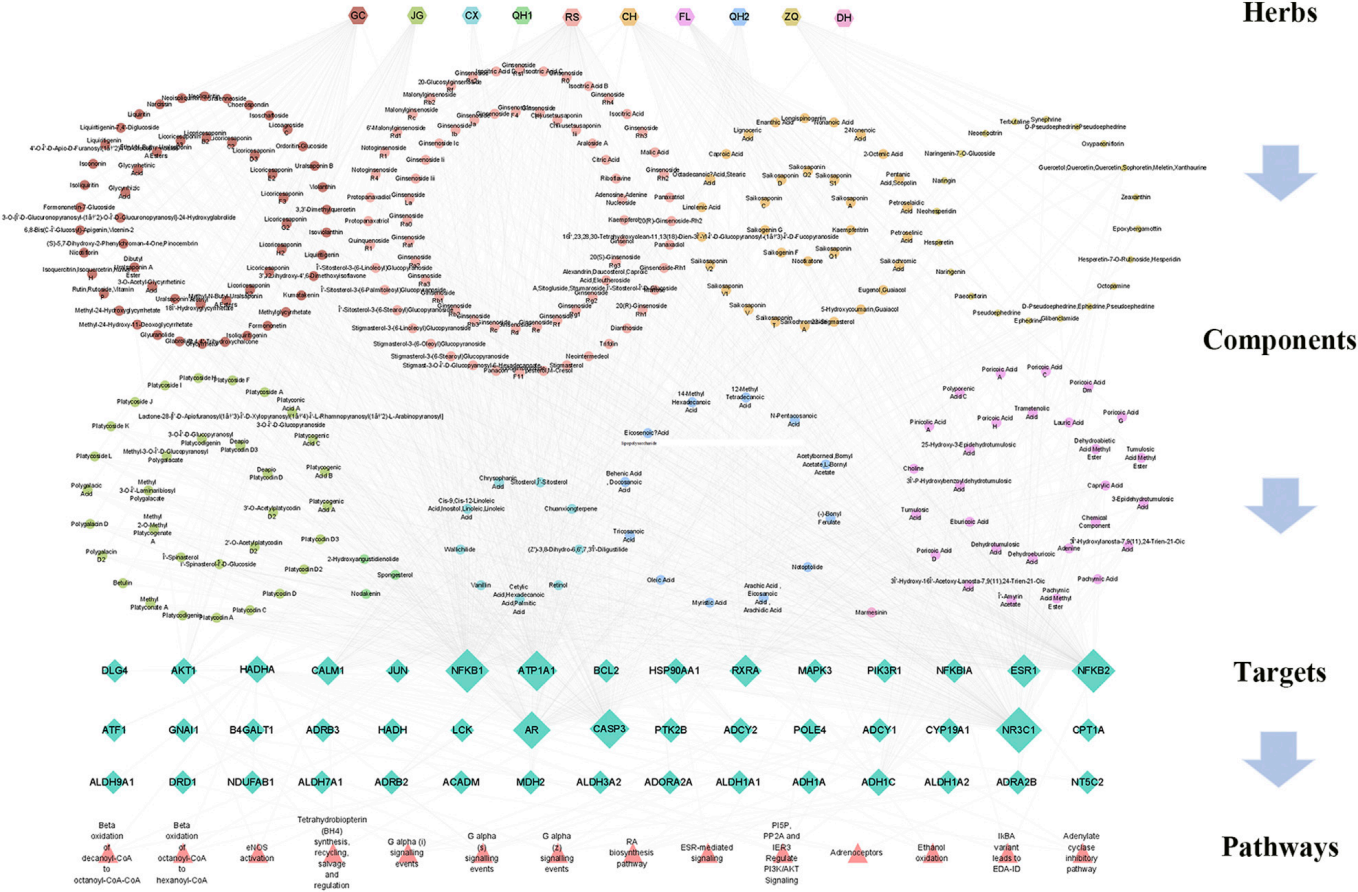
Correlation among the chemical components, candidate targets, and involved pathways of RSBDP. Hexagon nodes refer to medicinals, circular nodes refer to the major constituents in the 10 medicinals, diamond nodes refer to putative targets, and triangle nodes refer to the putative gene enrichment pathways.

To illustrate the major mechanism of RSBDP in the treatment of UC, an interaction network of medicine–target–disease was constructed based on the interactions among two gene sets *via* the TCM Association Network Mining module of TCMIP, and the topological network features were calculated automatically by TCMIP (nodes’ information are provided in the Supplementary Table S5). To determine the hub nodes, which may have high values and perform essential functions, the values of the nodes were calculated within the putative RSBDP target and UC-related gene interaction network. Consequently, 45 hub notes were screened in the network, and their degree, betweenness, and closeness are greater than the median of the corresponding feature values of all nodes. The multidimensional network of active ingredients of medicinals, corresponding 45 key targets, and 27 pathways was constructed (Figure 4).

### 3.3 Gene ontology enrichment and reactome pathway analysis

**FIGURE 5:**
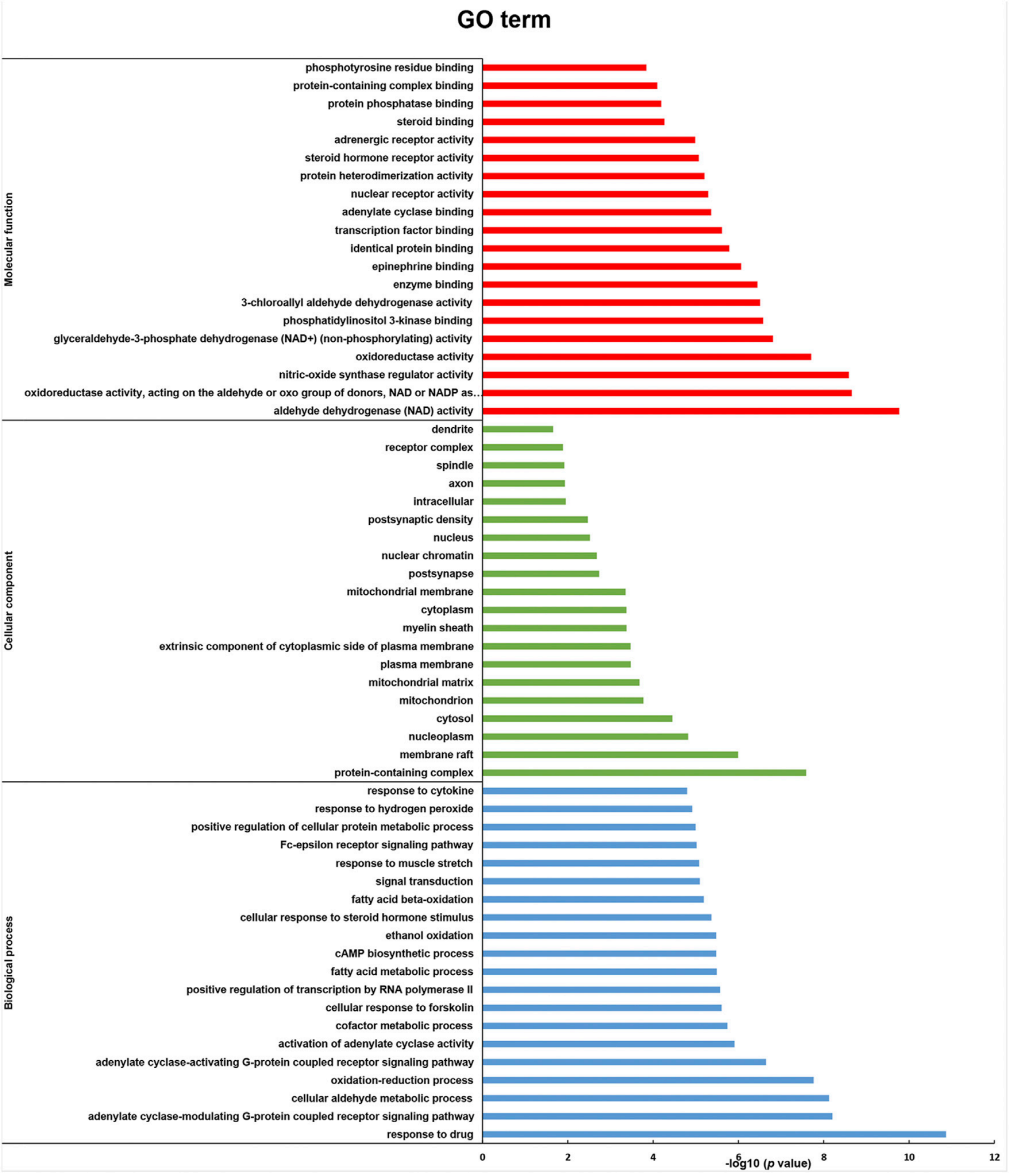
GO enrichment analysis (molecular function, cellular component, and biological process) of key effect genes of RSBDP on UC.

A total of 187 GO enrichment entries were obtained, including 104 biological processes (BP), 51 molecular functions (MF), and 32 cell compositions (CC). The visualization results of significantly enriched top 20 are shown in Figure 5. BP is mainly enriched in the response to cytokine, response to hydrogen peroxide, positive regulation of cellular protein metabolic process, Fc-epsilon receptor signaling pathway, and response to muscle stretch. MF is mainly enriched in phosphotyrosine residue binding, protein-containing complex binding, protein phosphatase binding, steroid binding, and adrenergic receptor activity. CC is mainly enriched in dendrite, receptor complex, spindle, axon, and intracellular components.

**FIGURE 6:**
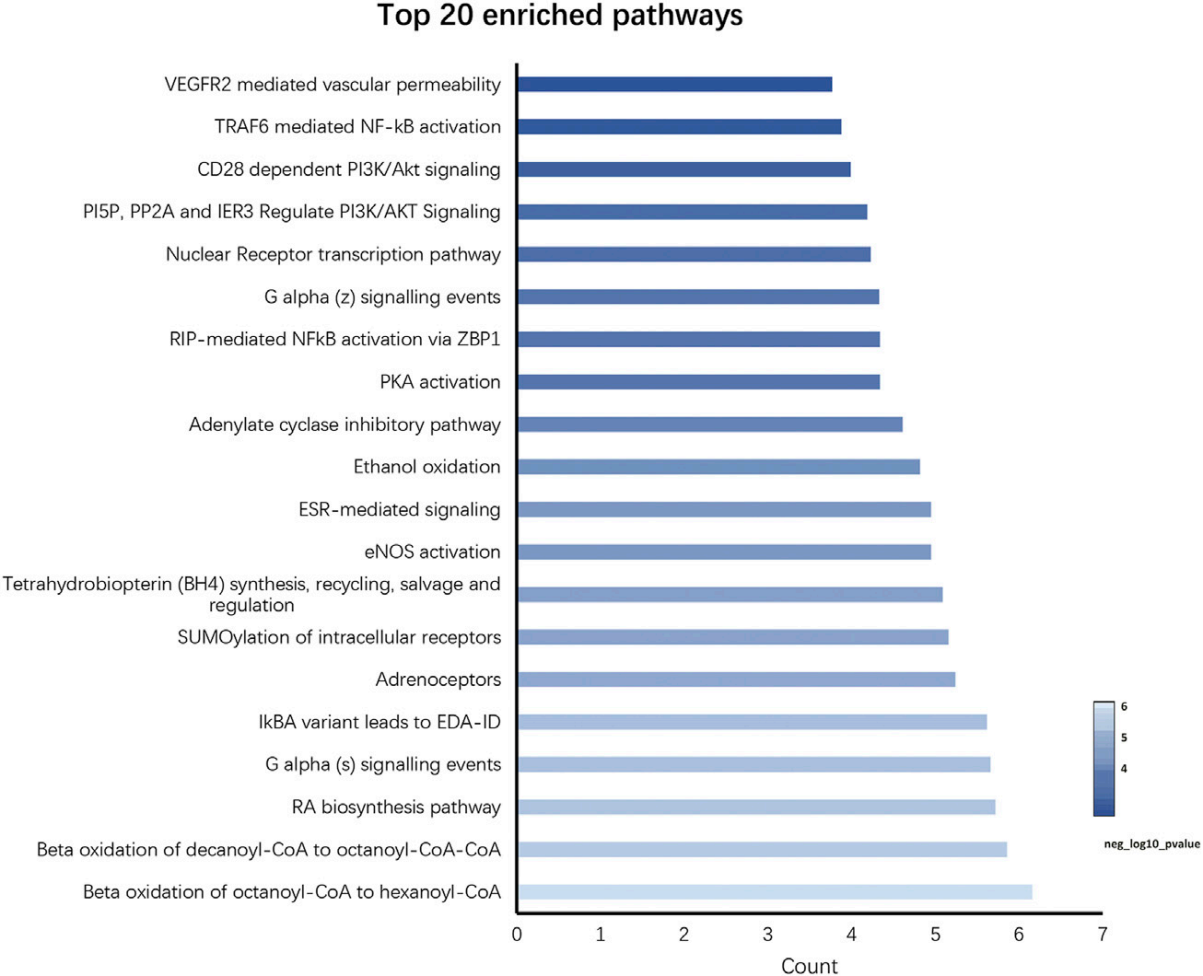
Reactome pathway analysis of key targets.

In total, 34 signaling pathways were screened (*p* < 0.05) by using the Reactome pathway database, and the visualization results of top 20 with significant enrichment are provided (Figure 6). The intervention effects mainly include the VEGFR2-mediated vascular permeability pathway, TRAF6-mediated NF-κB activation pathway, and CD28-dependent PI3K/Akt signaling pathway; PI5P, PP2A, and IER3 regulate the PI3K/Akt signaling pathway and the nuclear receptor transcription pathway. Following the Reactome pathway analysis, we established that PI3K/Akt/NF-κB is a crucial pathway, which is highly associated with the treatment of RSBDP.

### 3.4 Experimental validation

#### 3.4.1 The effect of Renshen Baidu powder on ulcerative colitis

**FIGURE 7:**
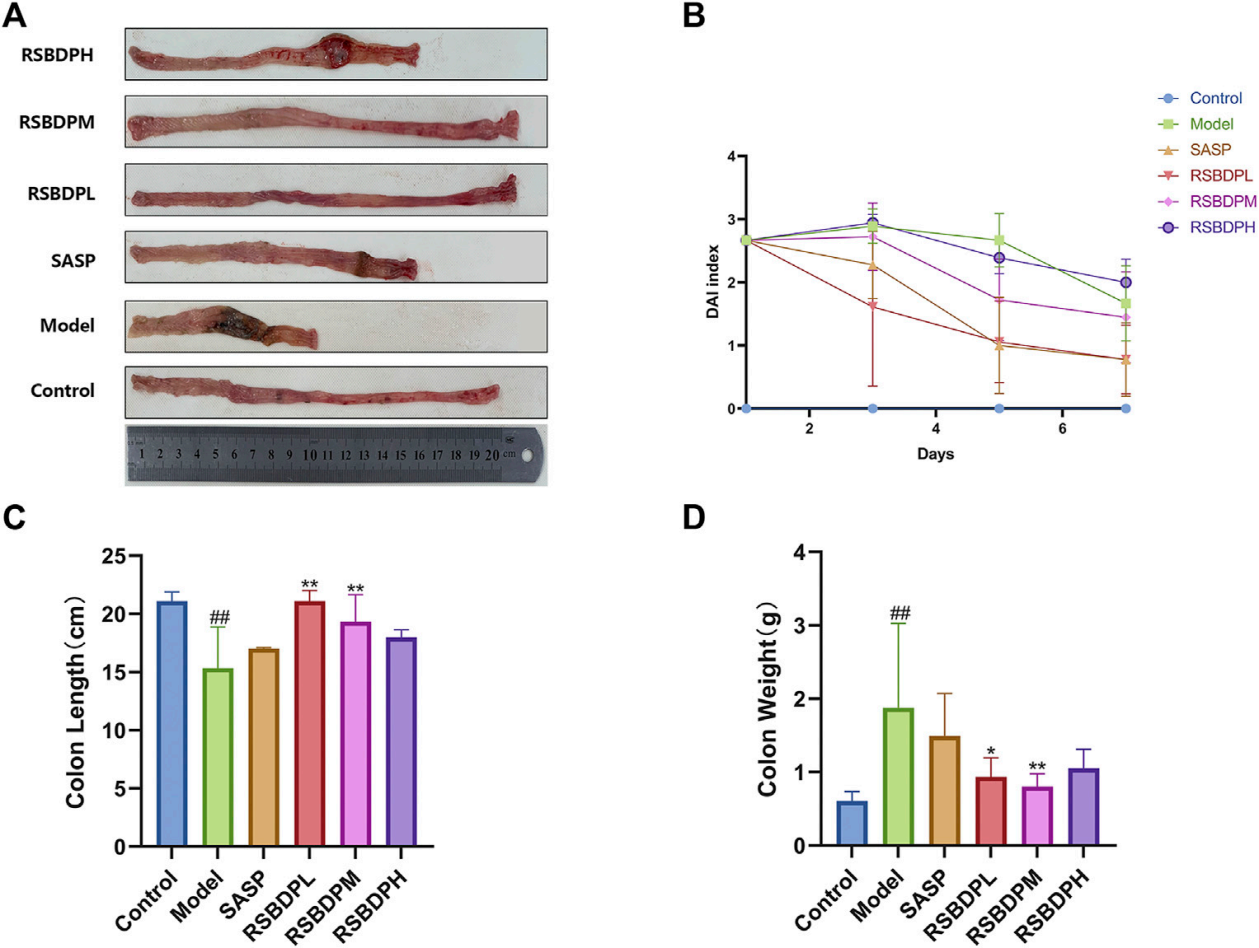
Effects of different doses of RSBDP and SASP on the pathogenic status, DAI score, colon weight, and colon length of TNBS-induced colitis in rats. **(A)** Pathogenic status; **(B)** DAI score; **(C)** weight of colons; **(D)** length of colons. Data are presented as $\bar{X}$ ± SEM (*n* = 6). #*p* < 0.05 and ##*p* < 0.01 versus control group; *p < 0.05 and **p < 0.01 versus model group.

The disease activity index (DAI) of the model group was significantly increased after TNBS administration (Figure 7A). The daily activity of rats in the model group decreased, appetite decreased, and hair color faded, accompanied with weight loss and loose stool. Compared with the control group, the DAI and weight of these rats was significantly decreased. The symptoms of the RSBDP group and the sulphasalazine group were milder than those of the model group. Moreover, the colon weight and colon length of the model group were significantly different from those of the control group. Compared with the control group, the colon weight of the model group was significantly increased, and the colon length was considerably shortened. Compared with the model group, the colon weight of rats in the RSBDP treatment group was significantly decreased, and the improvement was the most obvious in the medium-dose RSBDP group. TNBS led to shortened colon, and RSBDP treatment improved this phenomenon during the progression of colitis. The colon length of the RSBDP medium-dose group and RSBDP low-dose group was longer than that of the control group, and the improvement of the RSBDP high-dose group was the most obvious (Figures 7B,C).

#### 3.4.2 Histological observation and evaluation

**FIGURE 8:**
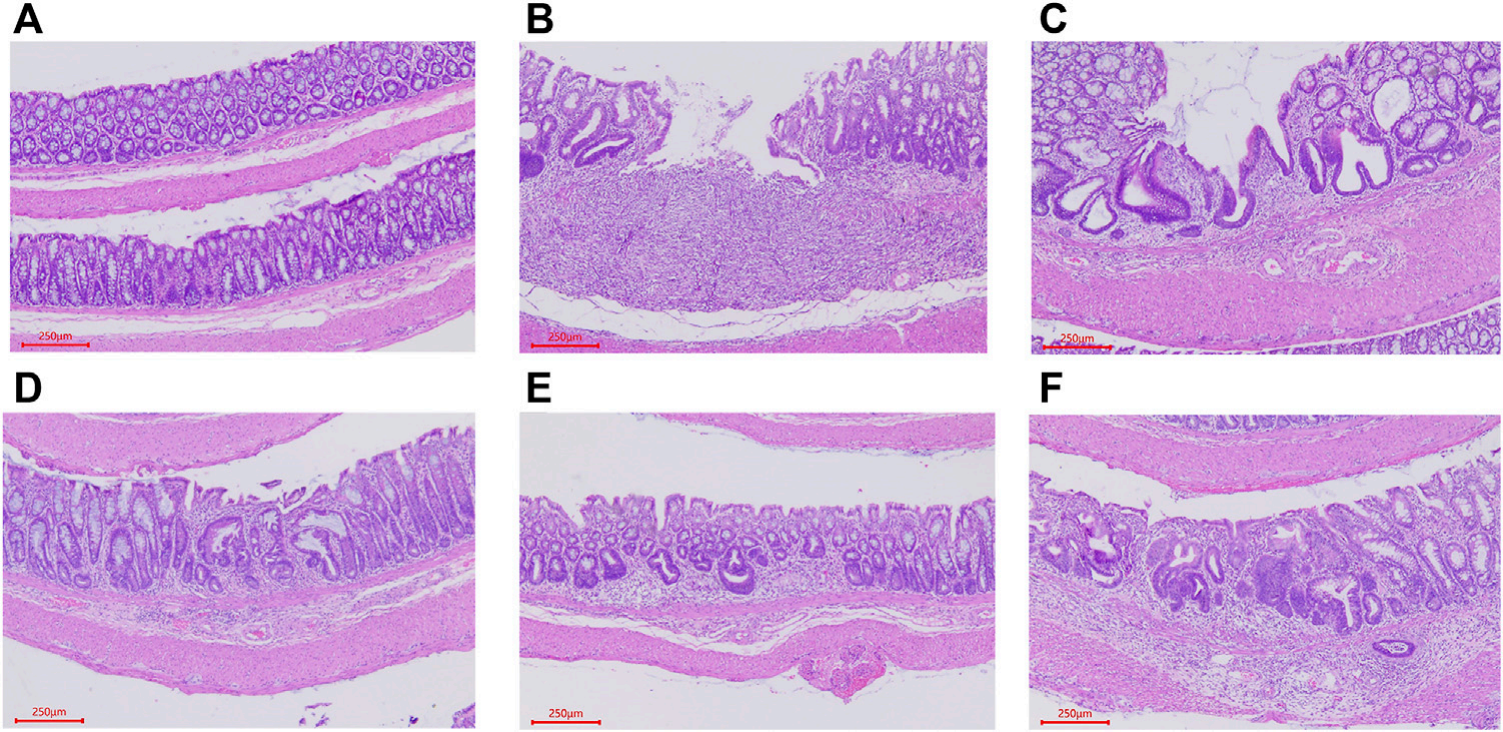
Representative H&E staining slices from colorectal sections, original magnification ×200. **(A)** Control group; **(B)** model group; **(C)** sulfasalazine group; **(D**–**F)**. RSBDPL, RSBDPM, REBDPH groups.

A histological analysis was performed to estimate the extent of damage to colon tissues. HE staining of the control group displayed intact surface epithelium, crypt, muscularis mucosa, and submucosa. In contrast, the histological analysis of distal colon of TNBS-treated rats showed significant mucosal ulceration and crypt destruction, accompanied by extensive involvement of mucosal and submucosal inflammatory cell infiltration. The treatment of RSBDP and sulphasalazine treatment groups alleviated intestinal wall ulcers, inflammatory cell infiltration, and crypt damage to varying degrees. Among them, the RSBDP low-dose group had the best protective effect on colon tissue damage. With the increase of the RSBDP dose, colon tissue damage did not decrease but increased (Figure 8). This may be related to the accumulation of the toxicity of traditional Chinese medicine (Zhang and Feng, 2019).

#### 3.4.3 Effect of trinitrobenzene sulfonic acid–induced pathological products

**FIGURE 9:**
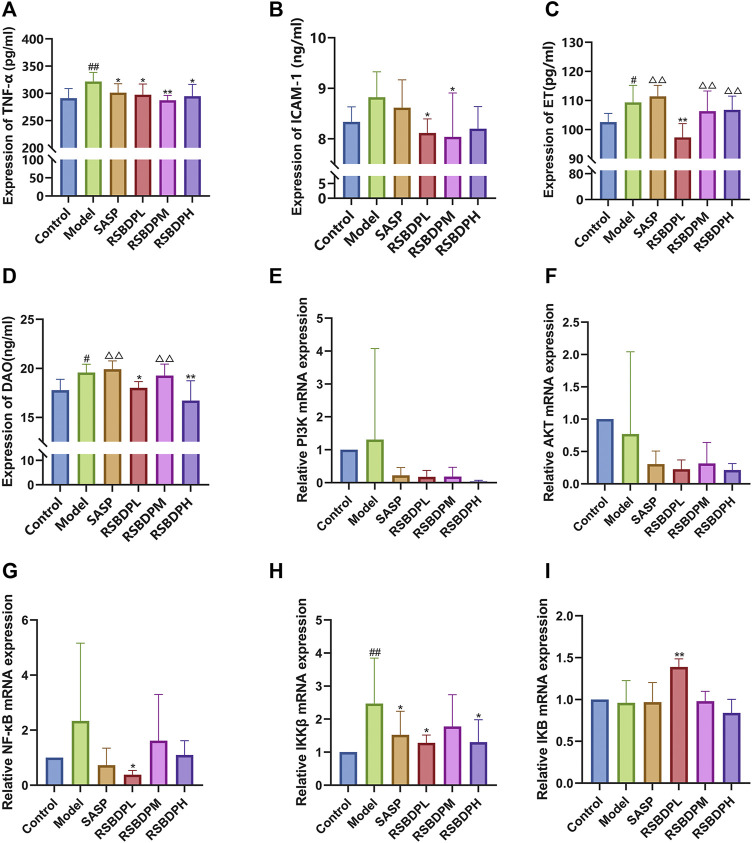
Effect of pathological products and PI3k, Akt, IKK, IκB, and NF-κB mRNA expressions in the colons of TNBS-induced colitis rats. **(A)** TNF-α; **(B)** ICAB-1; **(C)** ET; **(D)** DAO; **(E)** PI3K; **(F)** Akt; **(G)** NF-κB; **(H)** IKKβ; **(I)** IκB. Data are presented as $\bar{X}$ ± SEM (*n* = 6). #*p* < 0.05 and ##*p* < 0.01 versus control group; *p < 0.05 and **p < 0.01 versus model group; △*p* < 0.05 and △△*p* < 0.01 versus RSBDP low-dose group.

Inflammation is an integral part of UC-associated intestinal damage. Therefore, whether excessive inflammation occurred is the first to determine. First, the levels of inflammatory factors TNF-α in blood was detected (Figure 9A). Compared with the control group, the level of TNF-α production in the TNBS-induced colitis group was significantly increased, but this pathogenically increase were reversed by the treatment of RSBDP. This result demonstrated that RSBDP reverses the level of pro-inflammatory cytokine in colitis.

The accumulation of neutrophils is one of the most prominent histological features of the injured colon (Bradley et al., 1982). The presence of neutrophils within crypts and the mucosal layer of the colon are directly related to disease activity in UC (Nakajima et al., 1995; Naito et al., 2007). Adhesion molecules, including intercellular adhesion molecule-1 (ICAM-1), play a crucial role in the extravasation of neutrophils and adhesion from vascular lumen to inflammatory areas. Compared with the control group, the level of ICAM-1 production in the RSBDP treatment group was decreased and that in the low-dose group was significantly decreased (Figure 9B).

A healthy intestinal barrier can protect endotoxin (ET) from entering the blood circulation, while a dysfunctional intestinal barrier cannot prevent ET from entering the blood circulation through the intestinal mucosa, resulting in endotoxemia (van Deventer et al., 1988; Gardiner et al., 1995). Diamine oxidase (DAO) exists in the mucosa or villi of mammals, usually abundant in intestinal mucosa, kidney, and placental tissues, but rarely in serum (Honzawa et al., 2011). After intestinal mucosal epithelial damage, the cytoplasm DAO can be released into blood circulation (Fogel and Lewinski, 2006; Honzawa et al., 2011). Therefore, the serum ET level and DAO activity are ideal indicators to reflect the structure and function of the intestinal mucosa. Compared with the control group, the ET and serum DAO levels in the model group were significantly increased. Compared with the model group, the levels of DAO and ET in the RSBDP treatment group were decreased. Among them, RSBDP low-dose group had more obvious regulation on the ET level, while the high-dose group had more obvious regulation on the serum DAO level (Figures 9C,D).

#### 3.4.4 The effect of Renshen Baidu powder on the PI3k/Akt/NF-κB signaling pathway

**FIGURE 10:**
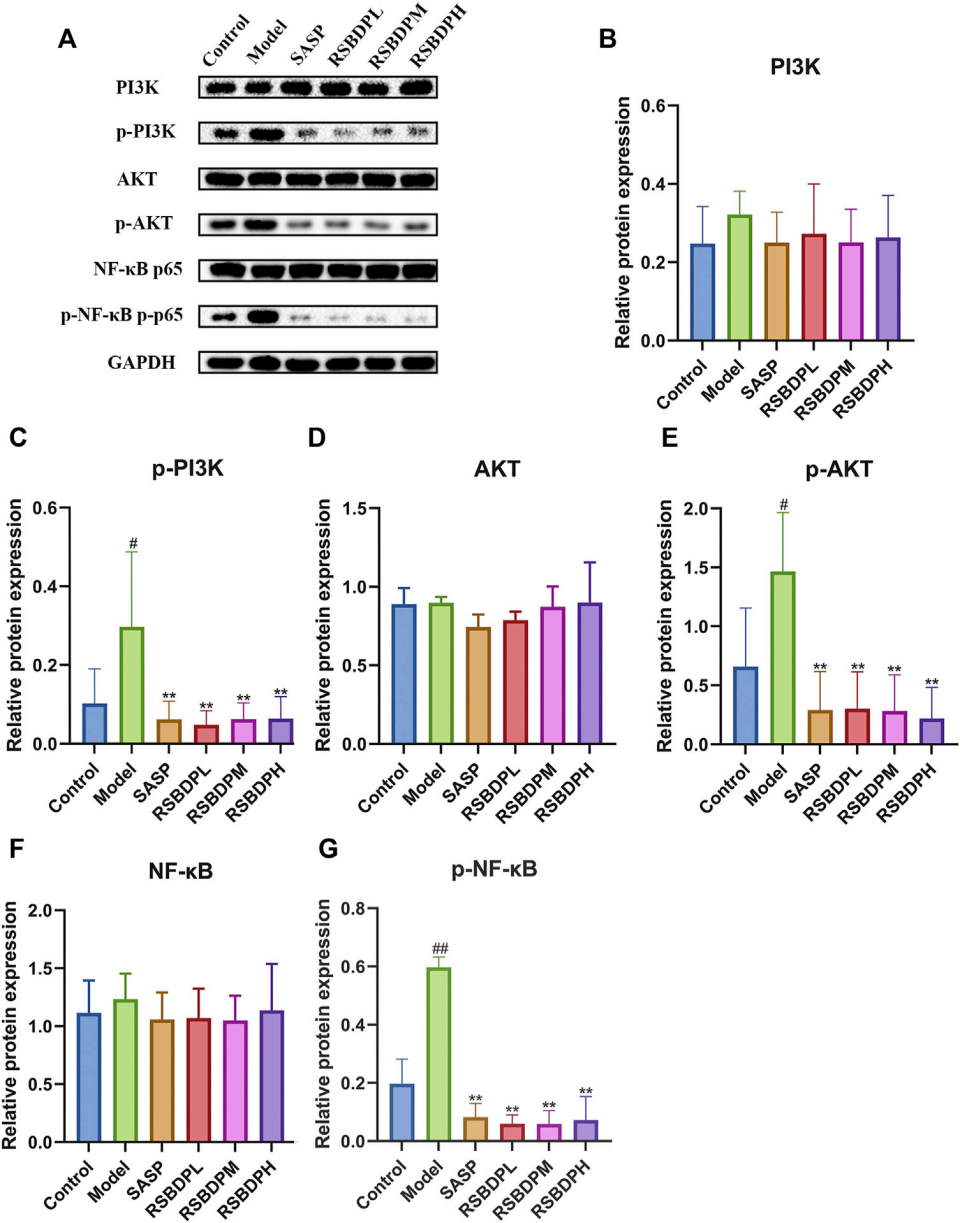
Effects of RSBDP on the expression of the PI3K/Akt/NF-κB signaling pathway–related proteins in TNBS-induced colitis rats **(A)**. The blot of PI3K, p-PI3K, Akt, p-Akt, NF-κB, and p-NF-κB. **(B)** PI3K; **(C)** p-PI3K; **(D)** Akt; **(E)** p-Akt; **(F)** NF-κB; **(G)** p-NF-κB. Data are presented as $\bar{\;X}$ ± SEM. #*p* < 0.05 and ##*p* < 0.01 versus control group; *p < 0.05 and **p < 0.01 versus model group.

The signaling pathway–related genes and proteins were investigated to further characterize the anti-inflammatory mechanism of RSBDP by activating the PI3K/Akt/NF-κB signaling pathway *via* PCR and Western blot analysis. The mRNA expression of PI3K and NF-κB in the model group was upregulated while that of Akt and IκB was downregulated (Figures 9E–I). Nevertheless, the changes in the four groups were not significant. Compared with the control group, the transcription of IKKβ [IKKβ is a subunit of the IKK complex, which can represent IKK (Flores et al., 2017)] in the model group was significantly increased. RSBDP significantly inhibited the mRNA expression of IKK and upregulated mRNA expression of IκB considerably. Moreover, the effect on the low-dose RSBDP group was more prominent than other groups. A Western blot analysis showed that compared with the control group, the total protein expressions of PI3K, Akt, and NF-κB in the rats from the model group showed no significant changes (Figures 10B,D,F). In addition, the contents of p-PI3K, p-Akt, and p-NF-κB were significantly increased (Figures 10C,E,G). Compared with the model group, the RSBDP high-, medium-, and low-dose groups could substantially reduce the expression of p-PI3K, p-Akt, and p-NF-κB proteins (Figures 10C,E,G). The results demonstrated that RSBDP can inhibit the PI3K/Akt/NF-κB signaling pathway from phosphorylation suppression and downregulation of activating factor IKKβ.

## 4 Discussion

UC is a subtype of IBD characterized by widespread colonic mucosal inflammation (Ordás et al., 2012). Due to the intricacy of the mechanism involved in the occurrence and development of UC, the current single targeted pharmacological treatment cannot achieve satisfactory results, and the long-term application of the drugs will result in certain side effects (Xiao et al., 2016; Eisenstein, 2018; Feuerstein et al., 2019). RSBDP is a well-known traditional Chinese medicinal formula, which has been proved to have a positive effect on UC (Xiong et al., 2021; Chen et al., 2019). However, the mechanisms of RSBDP against inflammation has not been mentioned in depth.

In this study, RSBDP shows predominant treatment on experimental colitis. In addition, network analysis results suggest that RSBDP may act on the targets such as nuclear receptor subfamily three group C member 1 (NR3C1), NF-κB (NFKB1 and NFKB2), and ATPase Na+/K + transporting subunit-α-1 (ATP1A1) through components such as caprylic Acid, glycyrrhetinic acid, eburicoic acid, nodakenin, and stigmasterol. Moreover, PI3K/Akt/NF-κB is considered to be a significantly related signaling pathway. The PI3K/Akt signaling pathway has been proved to increase β-catenin, resulting in the distortion of the crypt structure in chronic ulcerative colitis (Lee et al., 2010). NF-κB was reported that can attract to the iNOS promoter *in vivo via* an IKKβ-mediated pathway in UC (Andresen et al., 2005). As a classical inflammatory pathway, PI3K/Akt/NF-κB signaling pathway plays a critical role in the pathogenesis of UC (Bradley et al., 1982). PI3K can phosphorylate Akt, hence activating NF-κB and increasing the production of pro-inflammatory cytokines such as IL-6, IL-1β, and TNF-α. Therefore, PI3K, Akt, and NF-κB have been identified as potential therapeutic targets. On the other hand, activated NF-κB can also promote the activation of the NLRP3 inflammatory body, resulting in a cascade of inflammatory responses and mucosal damage (Kazi and Qian, 2009; Wu et al., 2013; Setia et al., 2014; Tian et al., 2016; Zhong et al., 2016; Jeengar et al., 2017). Inhibition of PI3K/Akt signaling pathway has been found in studies to reduce inflammatory cell damage, protect human intestinal cells, and hence treat UC (Qiao et al., 2018). Therefore, inhibition of PI3K/Akt/NF-κB signaling pathway may be the key mechanism for RSBDP to attenuate the inflammatory response of UC.

In order to clarify the chemical constituents of RSBDP aqueous extract, the aqueous extract of RSBDP was subjected to component analysis. The results indicated that the aqueous extract contained ginsenoside Rf, ginsenoside Rg2, notoginsenoside R2, and saikosaponin D, which were found to be anti-inflammatory (Li et al., 2020; Zhu et al., 2021). Then, the vivo experiment was conducted with SD rats to inhibit the PI3K/Akt/NF-κB signaling pathway. First, RSBDP is capable of reversing the symptoms that appeared in the model group. Meanwhile, RSBDP can downregulate the level of TNF-α in peripheral blood, thereby inhibiting the inflammatory response in peripheral blood. In addition, the expression levels of ICAM-1, DAO, and ET in peripheral blood were decreased. All these beneficial effects imply that RSBDP may be able to reduce inflammation by inhibiting the recruitment of pro-inflammatory cells while increasing intestinal permeability and therefore ameliorating pathological injury, which is consistent with prior research (Rana et al., 2014; Yan et al., 2020; Tatiya-Aphiradee et al., 2021). RSBDP could inhibit the development and expansion of ulcer foci in colon tissue, attenuate crypt damage, and alleviate inflammatory cell infiltration, thereby improving UC pathological injury. In addition, the therapeutic effect of RSBDPH is worse than that of RSBDPM and RSBDPL groups. This appearance demonstrates that other factors might influence the therapeutic effect, which is needed to be further investigated. As noted in the network analysis, proteins associated with PI3K/Akt/NF-κB signaling pathways were investigated. As described in the data, the transcription of PI3K and Akt did not show significant differences among the groups. Only low-dose RSBDP could downregulated the expression of NF-κB mRNA. Thus, RSBDP does not inhibit PI3K/Akt/NF-κB signaling pathway by interfering with the process of mRNA transcription. It was also discovered that although RSBDP had no effect on the total protein level of the pathway, the formula inhibited the activation of the pathway by inhibiting the phosphorylation of PI3K, Akt, and NF-κB.

**FIGURE 11:**
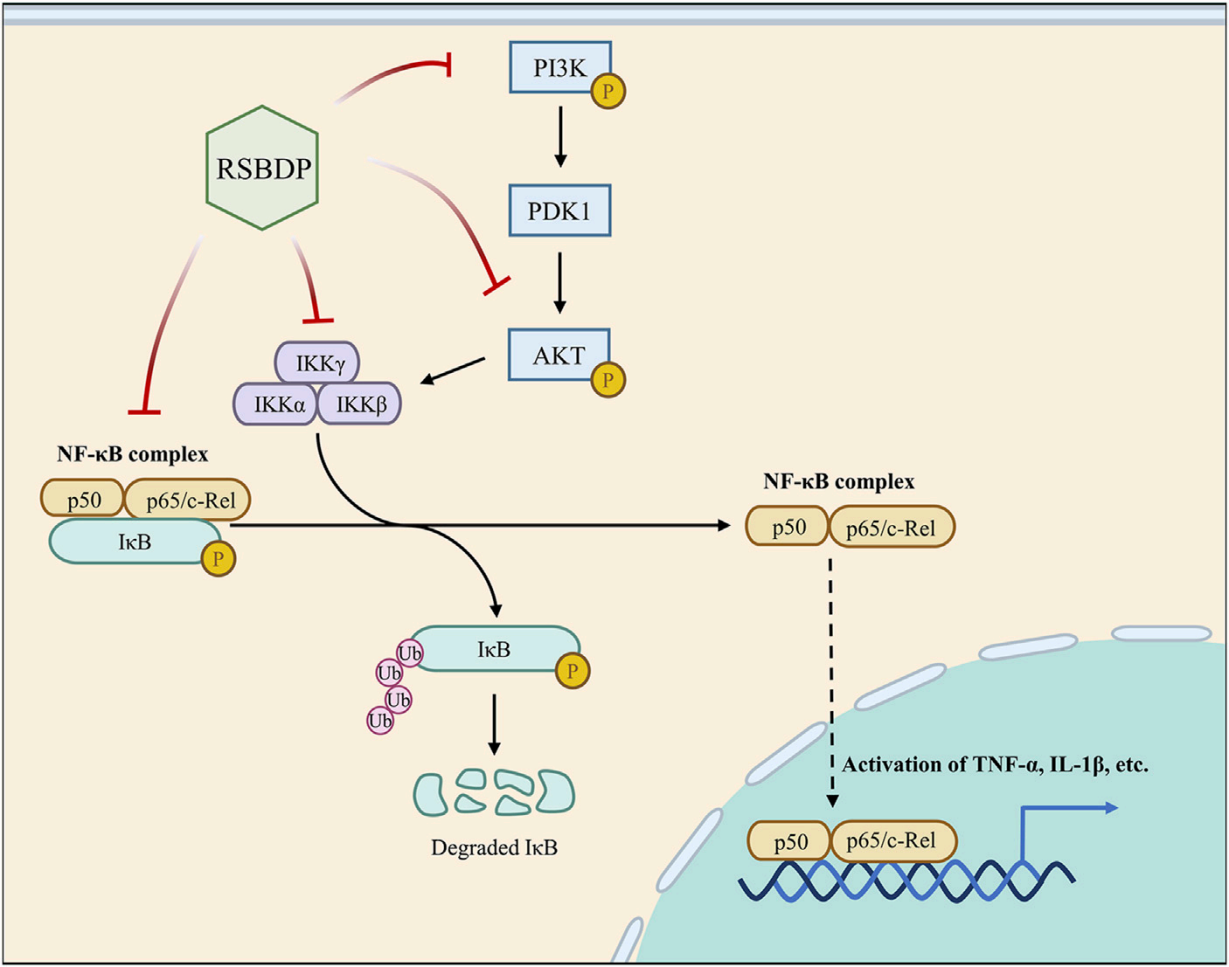
Major pathways of RSBDP treating UC. In a PDK1-dependent way, activated PI3K stimulates Akt activation. IKK is a downstream signal transduction pathway that can be regulated by activated Akt. The IKK complex is composed of the two catalytic subunits IKK-α and IKK-β and the regulatory subunit IKK-γ. The activated IKK phosphorylated its substrate IκB so that the NF-κB subunit p65 and p50 were transferred from cytoplasm to nucleus to activate NF-κB. The activated NF-κB can promote pro-inflammatory cytokines and increase the levels of cytokines, such as IL-6 and TNF-α. In this research, RSBDP can inhibit the phosphorylation of PI3K, Akt, and NF-κB while upregulating the NF-κB inhibitor protein IκB and downregulating the activator protein IKK, thus blocking the activation of the PI3K/Akt/NF-κB signaling pathway and treating UC.

Also, IκB, as an inhibitory protein of NF-κB, binds to the active site of the NF-κB protein during NF-κB quiescence. IKK protein moiety, as the activator of IκB, can enhance NF-κB phosphorylation by enabling IκB detachment from NF-κB once it is phosphorylated. In this study, the transcription of IκB and IKK were also regulated by RSBDP. The data reflected that RSBDP operated as an inhibitor of NF-κB activation by increasing IκB expression and decreasing IKK expression. In brief, RSBDP inhibited the phosphorylation of PI3K, Akt and NF-κB while decreasing IKK production and boosting IκB (Figure 11). This appearance is consistent with the multi-component and multi-target properties of RSBDP.

The therapeutic effect and mechanism of RSBDP aqueous extracts are readily apparent. To determine the major active components of RSBDP, the active ingredients extracted during the network analysis were compared with the results of mass spectrometry component analysis. The results indicated that only those compounds that could not meet both OB and DL were found in both RSBDP aqueous extract and network pharmacological analysis (Table 1). These compounds were all from the three medicinals RS, CH, and GC, suggesting that RS, CH, and GC may be the key medicinal on UC by RSBDP. Ginsenosides such as Rb1, Rb2, Rc, Rd, Re, Rf, Rg1, and Ro have been demonstrated in studies to restore intestinal microflora diversity and promote the reproduction of gut probiotics such as *Akkermansia*, *Bifidobacterium*, and *Lactobacillus* (Zhu et al., 2021).

**TABLE 1:** Compounds in mass spectrometry and the TCMIP analysis.

No.	RT (min)	Formula	Molecular Weight	Addition mode	Error in ppm	Molecular Weight	Addition mode	Error in ppm	Compound name	Source	OB (%)	DL
1	23.39	C_47_H_80_O_18_	NA	NA	NA	977.5354	M + HCOO	3.4	Notoginsenoside R1	RS	4.27	0.13
2	24.8	C_42_H_72_O_14_	823.4783	M + Na	−4.5	845.4903	M + HCOO	0.5	Ginsenoside Rg1	RS	—	—
3	24.98	C_48_H_82_O_18_	969.5427	M + Na	2.9	991.5481	M + HCOO	0.3	Ginsenoside Re	RS	4.27	0.12
4	32.32	C_42_H_72_O_14_	NA	NA	NA	845.4903	M + COO	0.5	Ginsenoside Rf	RS	17.74	0.24
5	33.74	C_41_H_70_O_13_	NA	NA	NA	815.4822	M + HCOO	3.6	Notoginsenoside R2	RS	17.74	0.28
6	35.67	C_42_H_72_O_13_	NA	NA	NA	829.4908	M + HCOO	−4.9	Ginsenoside Rg2	RS	8.32	0.25
7	37.47	C_48_H_78_O_17_	NA	NA	NA	925.5199	M-H	4.1	Saikosaponin c	CH	5.12	0.05
8	38.4	C_54_H_92_O_23_	NA	NA	NA	1153.5956	M + HCOO	−4.3	Ginsenoside Rb1	RS	6.24	0.04
9	39.39	C_48_H_76_O_19_	979.4927	M + Na	5	955.4900	M-H	−0.3	Ginsenoside Ro	RS	1.98	0.05
10	39.71	C_53_H_90_O_22_	NA	NA	NA	1123.5884	M + COO	−1.4	Ginsenoside Rc	RS	8.16	0.04
11	41.16	C_53_H_90_O_22_	NA	NA	NA	1123.5884	M + COO	−1.4	Ginsenoside Rb2	RS	6.02	0.04
12	42.09	C_42_H_62_O_16_	823.4076	M + H	−4.9	821.3920	M-H	−1.2	Glycyrrhizic acid	GC	19.62	0.11
13	43.76	C_48_H_82_O_18_	NA	NA	2.9	991.5481	M + HCOO	0.3	Ginsenoside Rd	RS	—	—
14	45.56	C_42_H_62_O_16_	823.4076	M + H	−4.9	821.3920	M-H	−1.2	Uralsaponin B	GC	7.92	0.11
15	46.4	C_42_H_68_O_13_	NA	NA	NA	779.4589	M-H	0.6	Saikosaponin d	CH	34.39	0.09

Retention time (RT); oral bioavailability (OB); drug-likeness (DL); not applicable (NA).

In addition, the metabolite compound K of ginsenosides has a higher bioavailability and biological activity than the prototype compound, and the primary component of the metabolite compound K has a therapeutic effect (Mathiyalagan et al., 2014). This condition reveals that a variety of active ingredients in RSBDP cannot be directly absorbed to treat UC, but are processed by intestinal microorganisms to produce therapeutic effects. Furthermore, ginseng and its extract also have antibacterial effects, which can affect the permeability of bacterial cell membranes, cell walls and the synthesis process of proteins and nucleic acids, and directly treat local ulcer foci in the intestine (Kachur and Suntres, 2016). At the same time, those active ingredients (e.g., saikosaponin D) with low DL and high OB and are exist in RSBDP aqueous extracts. Saikosaponin D has been demonstrated to be effective in treating experimental colitis by reducing inflammation, preserving intestinal barrier, and managing intestinal flora (Li et al., 2020). It verifies that low DL does not entirely mean that there are no positive effects. If the major medicinals and active ingredients can be further studied clearly, this will help to reduce the clinical application of RSBDP for the treatment of colon disease, thus avoiding unnecessary potential side effects or adverse reactions.

In conclusion, RSBDP can block the activation of PI3K/Akt/NF-κB signaling pathway *via* inhibiting the phosphorylation of PI3K, Akt, and NF-κB while upregulating the inhibitory protein IκB of NF-κB and downregulating the pathway activating protein IKK. This mechanism is evidential support for RSBDP intervention in the multi-targeted treatment for UC. Simultaneously, our data indicate that RS, CH, and GC may be the primary medicinal constituents of RSBDP, providing preclinical evidence for clinical use of RSBDP.

## 5 Conclusion

RSBDP can inhibit the phosphorylation of PI3K, Akt, and NF-κB; upregulate NF-κB inhibitor protein IκB; and downregulate the activator protein IKK, thus blocking the activation of the PI3K/Akt/NF-κB signaling pathway and treating UC. In addition, RS, CH, and GC may be the core medicinals of RSBDP.

## Data Availability

The original contributions presented in the study are included in the article/Supplementary materials; further inquiries can be directed to the corresponding authors.
