# Supplementary material for: Renshen Baidu powder protects ulcerative colitis via inhibiting the PI3K/Akt/NF-κB signaling pathway
Source: Front Pharmacol. 2022 Aug 10;13:880589. doi: 10.3389/fphar.2022.880589 (PMC9399752; doi:10.3389/fphar.2022.880589)
Supplement: Supplementary file 1 [file DataSheet1.ZIP › Supplementary Material/Supplementary Table 1, 2.docx]

Supplementary Material

# Supplementary Table

## Supplementary Table 1

| No. | RT (min) | Formula | Molecular weight | Addition mode | Error in ppm | Molecular weight | Addition mode | Error in ppm | Compound Name | Source |
| --- | --- | --- | --- | --- | --- | --- | --- | --- | --- | --- |
| 1 | 1.6 | C9H11NO2 | 166.0867 | M+H | -0.6 | 164.0718 | M-H | 3.7 | Phenylalanine | ZQ |
| 2 | 1.77 | C14H26O10 | 377.1409 | M+Na | -4 | 353.1453 | M-H | 1.4 | 2-{(6-Ο-[β-D-apiofuranosyl]-βD-glucopyranosyl)  oxy}propane | QH2 |
| 3 | 2.46 | C16H18O9 | 355.1043 | M+H | 3.9 | 353.0888 | M-H | 4.2 | Neochlorogenic acid | CX |
| 4 | 2.46 | C13H14O9 | 337.0556 | M+Na | -1.2 | 313.0553 | M-H | -2.2 | 2-hydroxybenzoic acid glucuronide | ZQ |
| 5 | 3.06 | C11H12N2O2 | 205.0986 | M+H | 1.5 | 203.0823 | M-H | 1 | Tryptophan | All |
| 6 | 3.43 | C13H14O9 | NA | NA | NA | 313.0553 | M-H | -2.2 | 3-hydroxybenzoic acid glucuronide | ZQ |
| 7 | 3.85 | C13H14O9 | NA | NA | NA | 313.0553 | M-H | -2.2 | 4-hydroxybenzoic acid glucuronide | ZQ |
| 8 | 4.15 | C16H20O9 | 379.0974 | M+Na | -0.3 | 355.1020 | M-H | -2.5 | Gentiopicrin | FL |
| 9 | 4.73 | C16H18O9 | 355.1043 | M+H | 3.9 | 353.0888 | M-H | 4.2 | Chlorogenic acid | CX |
| 10 | 5.12 | C20H24O12 | 479.1163 | M+Na | -0.4 | 455.1207 | M-H | 3.7 | Adicardin | DH |
| 11 | 5.71 | C16H18O9 | 355.1043 | M+H | 3.9 | 353.0888 | M-H | NA | Cryptochlorogenic acid | CX |
| 12 | 6.2 | C16H22O9 | NA | NA | NA | 357.1179 | M-H | -2 | methyl 3-(2-O-â-D-glucopyranosyl-4-  hydroxyphenyl)-propanoate | QH1 |
| 13 | 6.67 | C15H26O10 | NA | NA | NA | 365.1461 | M-H | 3.6 | 3‐{[3‐Hydroxy‐2‐(hydroxymethyl)‐2‐  methylpropanoyl]oxy}‐2‐({[3‐hydroxy‐2‐  (hydroxymethyl)‐2‐methylpropanoyl]oxy}methyl)‐2‐  methylpropanoic acid ouEuonyminol | ZQ |
| 14 | 6.87 | C16H18O10 | 393.0811 | M+Na | 3.3 | 369.0839 | M-H | 4.6 | Fraxin | QH2 |
| 15 | 7.63 | C21H28O11 | 479.1548 | M+Na | 4 | 455.1543 | M-H | -2.2 | 6-[(1R,2R)-1, 2-dihydroxy-3-b-D-glucosyloxy  -3-methylbutyl]-7-methoxycoumarin | DH |
| 16 | 8.34 | C20H26O13 | 497.1246 | M+Na | -5 | NA | NA | NA | Quercetin dimethylether | ZQ |
| 17 | 9.43 | C17H20O9 | 369.1194 | M+H | 2.2 | 367.1026 | M-H | -0.8 | Feruloylquinic acid | CX |
| 18 | 9.75 | C17H20O9 | 391.1007 | M+Na | 0.5 | 367.1032 | M-H | 0.8 | Methyl chlorogenate | DH |
| 19 | 9.75 | C16H30O10 | 405.1747 | M+Na | 2.5 | 381.1753 | M-H | -2.1 | NA | NA |
| 20 | 9.8 | C27H32O15 | 595.1664 | M+H | 0.2 | 593.1519 | M-H | 2.2 | Lonicerin | ZQ |
| 21 | 10.52 | C20H22O11 | 461.1078 | M+Na | 3.9 | 437.1074 | M-H | -2.3 | Loquatoside | ZQ |
| 22 | 10.73 | C21H28O11 | 479.1548 | M+Na | 4 | 455.1543 | M-H | -2.2 | 6-[(1R,2R)-1, 2-dihydroxy-3-b-D-  glucosyloxy-3-methylbutyl]-7-  methoxycoumarin | DH |
| 23 | 11.84 | C20H24O10 | 447.1264 | M+Na | -0.7 | 423.1310 | M-H | 4.5 | Decuroside V | QH2 |
| 24 | 12.6 | C20H24O10 | 447.1264 | M+Na | -0.7 | 423.1310 | M-H | 4.5 | 10-O--D-glucopyranosyl (2R  3S)-3-hydroxynodakenetin | QH2 |
| 25 | 12.6 | C33H42O19 | 743.2401 | M+H | 0.3 | 741.2142 | M-H | 0 | Naringenin -7-O-triglycoside | ZQ |
| 26 | 12.71 | C21H22O9 | 441.115 | M+Na | -2.7 | 417.1187 | M-H | 0.2 | Liquiritin | Liquiritin |
| 27 | 13.03 | C21H22O9 | 441.115 | M+Na | -2.7 | 417.1187 | M-H | 0.2 | Isoliquiritin | Isoliquiritin |
| 28 | 13.38 | C20H24O10 | 447.1264 | M+Na | -0.7 | 423.1310 | M-H | 4.5 | Apterin | QH2 |
| 29 | 13.51 | C20H26O10 | 449.1446 | M+Na | 4.9 | 425.1458 | M-H | 2.4 | Praeroside VI | QH1 |
| 30 | 13.51 | C26H30O13 | 573.1575 | M+Na | -1.6 | 549.1600 | M-H | -1.5 | Liquiritigenin-4‘- apioside | GC |
| 31 | 13.83 | C26H30O13 | 573.1575 | M+Na | -1.6 | 549.1600 | M-H | -1.5 | Liquiritin apioside | GC |
| 32 | 14.11 | C33H42O19 | NA | NA | NA | 741.2252 | M-H | 1.3 | Naringin 4'-glucoside | ZQ |
| 33 | 14.86 | C27H32O15 | NA | NA | NA | 595.1677 | M-H | 2.4 | Eriodictyol-7-O-rutinoside (Eriocitrin) | ZQ |
| 34 | 14.98 | C27H32O15 | 597.1811 | M+H | -1.3 | 595.1677 | M-H | 2.4 | Eriodictyol-7-O-neohesperidoside (Neoeriocitrin) | ZQ |
| 35 | 15.38 | C20H24O9 | 431.1234 | M+Na | -1.9 | 453.1413 | M+HCOO | 3.5 | Columbianetin-​β-​D-​glucopyranoside | DH |
| 36 | 15.56 | C20H24O9 | 409.1490 | M+H | -2.2 | 453.1369 | M+HCOO | 2.6 | Nodakenin | DH |
| 37 | 16.66 | C27H32O14 | 581.1866 | M+H | -0.7 | 579.1703 | M-H | -1.9 | Naringenin-7-O-rutinoside (Narirutin) | ZQ |
| 38 | 16.8 | C21H20O10 | 435.1127 | M+H | -4.8 | 433.1140 | M-H | 1.2 | Naringenin-7-O-glucoside | ZQ |
| 39 | 17.03 | C20H24O9 | 431.1234 | M+Na | -1.9 | 453.1369 | M+HCOO | 2.6 | Marmesinin | DH |
| 40 | 17.64 | C27H32O14 | 581.1861 | M+H | -1.5 | 579.1703 | M-H | -1.9 | Naringenin-7-O-neohesperidoside (Naringin) | ZQ |
| 41 | 18.67 | C25H48O24 | NA | NA | NA | 755.2441 | M-H | 1.1 | Hesperetin-7-O-(2”,6”-di-O-rha)-glu | ZQ |
| 42 | 18.79 | C28H32O15 | 609.1826 | M+H | -0.7 | 607.1654 | M-H | -1.5 | Diosmin | ZQ |
| 43 | 18.79 | C28H34O15 | 611.1937 | M+H | -1.5 | 609.1835 | M-H | 2.6 | Hesperetin-7-O-rutinoside (Hesperidin) | ZQ |
| 44 | 19.27 | C34H44O20 | 773.2510 | M+H | 0.8 | NA | NA | NA | Alhagidin | QH2 |
| 45 | 19.76 | C28H34O15 | 611.1937 | M+H | -1.5 | 609.1835 | M-H | 2.6 | Hesperetin-7-O-neohesperidoside (Neohesperidin) | ZQ |
| 46 | 19.95 | C28H34O15 | 611.1977 | M+H | 0.2 | NA | NA | NA | Magnoloside E | ZQ |
| 47 | 20.33 | C34H40O21 | 785.2172 | M+H | 4.1 | 783.1970 | M-H | -1.8 | 4′-O-(2′′′-O-glucuronyl-glucosyl)-swertisin | QH2 |
| 48 | 20.33 | C21H22O9 | 441.1150 | M+Na | -2.7 | 417.1187 | M-H | 0.2 | Neoisoliquiritin | Neoisoliquiritin |
| 49 | 20.6 | C14H14O4 | 247.0968 | M+H | -0.8 | NA | NA | NA | Nodakenetin | DH |
| 50 | 20.6 | C22H22O9 | 431.1337 | M+H | -1.2 | 475.1252 | M+HCOO | 2.5 | Ononin | CX |
| 51 | 22.67 | C48H82O19 | NA | NA | NA | 961.5370 | M-H | -0.2 | 20-O-Glucosylginsenoside Rf | RS |
| 52 | 23.06 | C35H36O15 | NA | NA | NA | 695.1998 | M-H | 3.2 | Liquiritin B | GC |
| 53 | 23.17 | C29H36O15 | NA | NA | NA | 669.2035 | M+HCOO | 0.6 | Isoacteoside | CH |
| 54 | 23.32 | C48H76O20 | NA | NA | NA | 1017.4914 | M+HCOO | 0.8 | 3b,16a,23,28-tetrahydroxyoleana-  11,13(18)-dien-30-oic acid 3-O-b-D-glucopyranosyl-(1→2)-b-D-glucopyranosyl-  (1→3)-b-Dfucopyranoside | CH |
| 55 | 23.39 | C47H80O18 | NA | NA | NA | 977.5354 | M+HCOO | 3.4 | Notoginsenoside R1 | RS |
| 56 | 23.39 | C42H66O15 | NA | NA | NA | 809.4335 | M-H | NA | Bupleuroside V | CH |
| 57 | 24.09 | C34H42O19 | NA | NA | NA | 753.2346 | M-H | -0.1 | Brutieridin | ZQ |
| 58 | 24.09 | C54H84O24 | NA | NA | NA | 1115.5223 | M-H | -4.6 | licorice-saponin O4 | GC |
| 59 | 24.8 | C42H72O14 | 823.4783 | M+Na | -4.5 | 845.4903 | M+HCOO | 0.5 | Ginsenoside Rg1 | RS |
| 60 | 24.98 | C48H82O18 | 969.5427 | M+Na | 2.9 | 991.5481 | M+HCOO | 0.3 | Ginsenoside Re | RS |
| 61 | 26.01 | C28H34O14 | 595.2008 | M+H | -3.2 | 593.1862 | M-H | -1.3 | Isosakuranetin-7-O-  neohesperidoside (Poncirin) | ZQ |
| 62 | 26.45 | C44H74O15 | NA | NA | 0.9 | 841.4940 | M-H | -1.1 | Yesanchinoside D | RS |
| 63 | 27.39 | C20H24O7 | 377.1614 | M+H | 3.7 | NA | NA | NA | Angelol A | DH |
| 64 | 28.88 | C20H24O7 | 377.1614 | M+H | 3.7 | 421.1498 | M+HCOO | -0.2 | Angelol B | DH |
| 65 | 29.38 | C20H26O7 | 379.1768 | M+H | 2.9 | 423.1672 | M+HCOO | 4 | Anpubesol | DH |
| 66 | 29.58 | C20H26O7 | 379.1768 | M+H | 2.9 | NA | NA | NA | Angelol C | DH |
| 67 | 29.89 | C20H26O7 | 379.1768 | M+H | 2.9 | NA | NA | NA | Angelol I | DH |
| 68 | 29.89 | C52H84O24 | 1093.5396 | M+H | -3.2 | NA | NA | NA | Deapi-platycodin D | JG |
| 69 | 31.33 | C20H24O7 | 377.1614 | M+H | 3.7 | 421.1498 | M+HCOO | -0.2 | AngelolG | DH |
| 70 | 32.32 | C42H72O14 | NA | NA | NA | 845.4903 | M+COO | 0.5 | Ginsenoside Rf | RS |
| 71 | 32.56 | C20H24O7 | 377.1614 | M+H | 3.7 | 421.1498 | M+HCOO | -0.2 | Angelol D | DH |
| 72 | 32.8 | C20H26O7 | 379.1768 | M+H | 2.9 | 423.1672 | M+HCOO | 4 | Angelol L | DH |
| 73 | 32.96 | C48H72O21 | NA | NA | NA | 983.4529 | M-H | 4.2 | Glycyrrhizic acid A3 | GC |
| 74 | 33.41 | C44H64O18 | NA | NA | NA | 879.3978 | M-H | -4.1 | 22β-acetoxylglycyrrhizin | GC |
| 75 | 33.74 | C41H70O13 | NA | NA | NA | 815.4822 | M+HCOO | 3.6 | Notoginsenoside R2 | RS |
| 76 | 34.19 | C26H30O8 | 471.2023 | M+H | 0.8 | 515.1930 | M+HCOO | 2.5 | Limonin | ZQ |
| 77 | 34.19 | C44H74O15 | 865.4933 | M+Na | 0.9 | 841.4940 | M-H | -1.1 | Acetyl panajaponol A | RS |
| 78 | 34.62 | C18H34O5 | 353.2304 | M+Na | 0 | 329.2334 | M-H | 1.8 | 3′,4′-Dimethoxy quercetin | CH |
| 79 | 34.62 | C42H62O17 | 839.4045 | M+H | -2.4 | 837.3910 | M-H | 0.1 | Licorice-​saponin G2 | GC |
| 80 | 35.31 | C42H62O17 | NA | NA | NA | 837.3910 | M-H | 0.1 | Glycyrrhizic acid G2 | GC |
| 81 | 35.61 | C14H12O3 | 229.0862 | M+H | -1.3 | NA | NA | NA | Angenomalin | DH |
| 82 | 35.67 | C42H72O13 | NA | NA | NA | 829.4908 | M+HCOO | -4.9 | Ginsenoside Rg2 | RS |
| 83 | 36.75 | C42H64O16 | NA | NA | NA | 823.4105 | M-H | -1.3 | Uralsaponin C | GC |
| 84 | 37.47 | C48H78O17 | NA | NA | NA | 925.5199 | M-H | 4.1 | Saikosaponin c | CH |
| 85 | 38.37 | C42H62O17 | 839.4045 | M+H | -2.4 | 837.3910 | M-H | 0.1 | Uralsaponin N | GC |
| 86 | 38.4 | C54H92O23 | NA | NA | NA | 1153.5956 | M+HCOO | -4.3 | Ginsenoside Rb1 | RS |
| 87 | 38.53 | C48H80O17 | NA | NA | NA | 973.5344 | M+HCOO | -2.9 | Saikosaponin f | CH |
| 88 | 39.29 | C57H94O26 | NA | NA | NA | 1193.5961 | M-H | 0.5 | m-ginsenoside Rb1 | RS |
| 89 | 39.39 | C48H76O19 | 979.4927 | M+Na | 5 | 955.4900 | M-H | -0.3 | Ginsenoside Ro | RS |
| 90 | 39.61 | C42H62O17 | NA | NA | NA | 837.3910 | M-H | 0.1 | Macedonoside A | GC |
| 91 | 39.71 | C53H90O22 | NA | NA | NA | 1123.5884 | M+COO | -1.4 | Ginsenoside Rc | RS |
| 92 | 40.58 | C56H92O25 | NA | NA | NA | 1163.5858 | M-H | 0.8 | m-ginsenoside Rc | RS |
| 93 | 41.16 | C53H90O22 | NA | NA | NA | 1123.5884 | M+COO | -1.4 | Ginsenoside Rb2 | RS |
| 94 | 41.55 | C42H62O17 | 839.4045 | M+H | -2.4 | 837.3910 | M-H | 0.1 | Macedonoside B | GC |
| 95 | 41.92 | C56H92O25 | NA | NA | NA | 1163.5858 | M-H | 0.8 | m-ginsenoside Rb2 | RS |
| 96 | 41.92 | C48H74O21 | NA | NA | NA | 985.4626 | M-H | -1.8 | Yunganoside G1 | GC |
| 97 | 42.09 | C42H62O16 | 823.4076 | M+H | -4.9 | 821.3920 | M-H | -1.2 | Glycyrrhizic acid | GC |
| 98 | 43.63 | C42H64O15 | NA | NA | NA | 807.4122 | M-H | -1.4 | Glycyrrhizic acid B2 | GC |
| 99 | 43.76 | C48H82O18 | NA | NA | 2.9 | 991.5481 | M+HCOO | 0.3 | Ginsenoside Rd | RS |
| 100 | 44.52 | C51H84O21 | NA | NA | 2 | 1031.5478 | M-H | -4.8 | m-ginsenoside Rd | RS |
| 101 | 44.76 | C42H64O15 | NA | NA | NA | 807.4122 | M-H | -1.4 | Glycyrrhizic acid B2 | GC |
| 102 | 45.56 | C42H68O13 | 803.4580 | M+H | 2.7 | 779.4589 | M-H | 0.6 | Saikosaponin b1 | CH |
| 103 | 45.56 | C42H62O16 | 823.4076 | M+H | -4.9 | 821.3920 | M-H | -1.2 | Uralsaponin B | GC |
| 104 | 46.4 | C42H68O13 | NA | NA | NA | 779.4589 | M-H | 0.6 | Saikosaponin d | CH |
| 105 | 46.8 | C42H62O16 | NA | NA | NA | 821.3920 | M-H | -1.2 | Glycyrrhizic acid H2 | GC |
| 106 | 47.43 | C42H64O16 | NA | NA | NA | 823.4105 | M-H | -1.3 | Glycyrrhizic acid J2 | GC |
| 107 | 49.16 | C15H16O3 | 245.1174 | M+H | -1.6 | NA | NA | NA | Osthole | DH |

**Supplementary Figure 1.** Identification of chemical compounds in RSBDP by UPLC-Q-TOF/MS. Abbreviation in Table 1: Retention time (RT); not applicable (NA); *Panax ginseng* C. A. Mey. (Renshen, RS), *Poria cocos* (Schw.) Wolf (Fuling, FL), *Glycyrrhiza uralensis* Fisch. (Gancao, GC), *Citrus aurantium* L. (Zhiqiao, ZQ), *Platycodon grandiflorum* (Jacq.) A. DC. (Jiegeng, JG), *Bupleurum chinense* DC. (Chaihu, CH), *Peucedanum praeruptorum* Dunn (Qianhu, QH1), *Notopterygium incisum* Ting ex H. T. Chang (Qianghuo, QH2), *Angelica pubescens* Maxim.f. biserrata Shan et Yuan (Duhuo, DH), and *Ligusticum chuanxiong* Hort. (Chuanxiong, CX)

## Supplementary Table 2

| Fecal properties | Evaluation Criteria |
| --- | --- |
| Normal feces | Formed feces without blood |
| Poorly formed feces | Semi-formed, paste-like feces that do not adhere to the anus |
| Loose feces | Watery feces that adhere to the anus |

| Points | Weight loss score | Fecal property score | Occult blood/ Fecal blood score |
| --- | --- | --- | --- |
| 0 | No decrease; | Normal feces; | Negative; |
| 1 | Decrease < 5%; | Slightly poorly formed feces; | Negative; |
| 2 | Decrease from 5% to 10%; | Poorly formed feces; | Occult blood; |
| 3 | Decrease from 10% to 15%; | Loose feces; | Slightly fecal blood; |
| 4 | Decrease of ≥15%; | Loose feces with fecal blood; | Fecal blood with loose feces; |

**Supplementary Figure 2.** DAI scoring details.
